# Supplementary material for: Problematic Social Media Use and Cyber Aggression in Italian Adolescents: The Remarkable Role of Social Support
Source: Int J Environ Res Public Health. 2022 Aug 8;19(15):9763. doi: 10.3390/ijerph19159763 (PMC9367929; doi:10.3390/ijerph19159763)
Supplement: Supplementary file 1 [file ijerph-19-09763-s001.zip › ijerph-1822566-supplementary.pdf]

Supplementary material

Table S1 - Prevalence of cyberbullying victimization (absolute and relative frequencies) by age categories, gender, and Region (HBSC 2018 - Italy).

Cyberbullying victimization	11y.o. Boys		11y.o. Girls		13y.o. Boys		13y.o. Girls		15y.o. Boys		15y.o. Girls		All ages and genders	
	N	%	N	%	N	%	N	%	N	%	N	%	**N**	**%**
Piedmont	33	6.41	49	10.32	29	5.21	52	10.34	33	6.41	27	6.19	**223**	**7.43**
Aosta Valley	35	11.08	29	9.18	18	6.77	31	10.54	13	6.81	13	7.83	**139**	**8.97**
Lombardy	54	9.75	66	12.41	26	4.32	60	10.64	32	6.31	43	6.85	**281**	**8.3**
Veneto	85	8.44	99	9.78	72	7.06	113	11.04	49	5.75	62	7.98	**480**	**8.43**
Friuli Venezia Giulia	29	5.7	52	10.28	32	6	60	10.75	30	5.91	40	7.84	**243**	**7.78**
Liguria	47	9.61	50	10.16	36	7.26	50	12.35	24	5.18	42	10.05	**249**	**9.01**
Emilia Romagna	53	10.08	69	13.61	38	7.41	55	10.04	21	4.77	53	9.09	**289**	**9.27**
Tuscany	41	8.25	60	11.61	27	5.08	60	11.21	34	6.67	34	7.33	**256**	**8.38**
Umbria	17	8.9	24	13.33	17	8.06	30	15.15	9	4.11	25	9.09	**122**	**9.58**
Marche	42	7.87	52	10.81	35	6.36	44	8.82	29	6.14	50	9.16	**252**	**8.18**
Latium	41	8.06	51	10.39	25	5.2	50	10.18	27	5.01	36	8.39	**230**	**7.82**
Abruzzo	37	7.87	60	13.22	35	7.45	50	12.08	28	5.98	45	8.41	**255**	**9.07**
Molise	33	8.46	48	12.63	37	7.43	47	11.22	24	6.32	32	8.31	**221**	**9.01**
Campania	35	7.32	41	10.1	39	8.11	53	11.21	33	7.07	41	8.7	**242**	**8.72**
Apulia	49	10.38	57	11.95	44	8.07	54	10.74	30	5.86	33	6.09	**267**	**8.75**
Basilicata	24	8.3	26	10.2	12	4.14	39	11.96	13	4.73	13	6.4	**127**	**7.75**
Calabria	49	9.82	44	10.02	45	8.41	60	11.32	34	7.11	32	6.12	**264**	**8.79**
Sicily	46	10.24	43	10.12	38	7.68	56	11.59	36	6.57	46	10.09	**265**	**9.28**
Sardinia	24	9.38	32	11.47	23	6.63	27	8.82	13	7.6	10	5.52	**129**	**8.38**
Bolzano	25	6.79	28	7.37	25	6.2	35	8.31	15	4.79	21	5.71	**149**	**6.61**
Trento	43	9.03	22	4.77	38	7.16	52	10.12	18	3.71	41	7.74	**214**	**7.14**
**Total**	**842**	**8.6**	**1002**	**10.59**	**691**	**6.67**	**1078**	**10.77**	**545**	**5.85**	**739**	**7.84**	**4897**	**8.39**

Table S2 - Prevalence of cyberbullying perpetration (absolute and relative frequencies) by age categories, gender, and Region (HBSC 2018 - Italy).

Cyberbullying perpetration	11y.o. Boys		11y.o. Girls		13y.o. Boys		13y.o. Girls		15y.o. Boys		15y.o. Girls		All ages and genders	
	N	%	N	%	N	%	N	%	N	%	N	%	N	%
Piedmont	33	6.43	27	5.70	31	5.60	40	7.95	41	7.95	21	4.83	**193**	**6.44**
Aosta Valley	22	7.01	13	4.13	18	6.77	19	6.46	10	5.18	9	5.42	**91**	**5.88**
Lombardy	40	7.23	38	7.16	37	6.13	39	6.88	53	10.5	29	4.63	**236**	**6.97**
Veneto	71	7.08	64	6.33	76	7.47	76	7.43	89	10.48	52	6.66	**428**	**7.53**
Friuli Venezia Giulia	19	3.73	27	5.35	30	5.63	40	7.14	42	8.33	20	3.91	**178**	**5.70**
Liguria	31	6.35	37	7.52	37	7.49	31	7.62	28	6.06	33	7.89	**197**	**7.14**
Emilia Romagna	27	5.14	27	5.33	38	7.41	38	6.91	25	5.69	41	7.06	**196**	**6.29**
Tuscany	33	6.64	30	5.80	40	7.48	37	6.93	45	8.81	36	7.74	**221**	**7.22**
Umbria	10	5.24	9	5.00	11	5.19	14	7.07	17	7.73	32	11.64	**93**	**7.29**
Marche	35	6.54	26	5.42	43	7.82	35	7.04	38	8.07	34	6.24	**211**	**6.86**
Latium	31	6.08	32	6.50	25	5.19	29	5.92	58	10.76	41	9.53	**216**	**7.34**
Abruzzo	37	7.81	33	7.30	35	7.38	26	6.28	37	7.91	36	6.72	**204**	**7.24**
Molise	24	6.19	21	5.50	42	8.42	31	7.40	29	7.61	24	6.23	**171**	**6.97**
Campania	31	6.49	15	3.69	38	7.92	48	10.19	37	7.91	31	6.60	**200**	**7.21**
Apulia	38	8.07	33	6.89	44	8.04	44	8.75	38	7.42	39	7.21	**236**	**7.73**
Basilicata	19	6.53	13	5.14	16	5.48	30	9.26	22	7.97	7	3.45	**107**	**6.53**
Calabria	40	8.05	21	4.81	40	7.49	43	8.11	46	9.68	41	7.81	**231**	**7.71**
Sicily	32	7.13	18	4.22	34	6.84	32	6.64	60	10.95	34	7.39	**210**	**7.33**
Sardinia	23	9.02	21	7.53	29	8.38	23	7.52	16	9.36	11	6.08	**123**	**8.00**
Bolzano	18	4.88	12	3.15	29	7.20	31	7.31	17	5.45	11	2.98	**118**	**5.23**
Trento	39	8.28	21	4.58	46	8.70	37	7.21	26	5.37	37	6.98	**206**	**6.90**
**Total**	**653**	**6.68**	**538**	**5.69**	**739**	**7.13**	**743**	**7.42**	**774**	**8.32**	**619**	**6.56**	**4066**	**6.97**

Table S3 - Prevalence of Problematic Social Media Use (PSMU), absolute and relative frequencies by age categories, gender, and Region (HBSC 2018 - Italy).

**Cyberbullying perpetration**	**11y.o. Boys**		**11y.o. Girls**		**13y.o. Boys**		**13y.o. Girls**		**15y.o. Boys**		**15y.o. Girls**		**All ages and genders**	
	N	%	N	%	N	%	N	%	N	%	N	%	**N**	**%**
Piedmont	36	7.76	36	8.09	34	6.37	65	13.18	20	3.98	39	9.11	**230**	**8.02**
Aosta Valley	13	4.55	15	5.08	15	5.86	31	10.76	9	4.89	14	8.70	**97**	**6.6**
Lombardy	50	9.94	49	10.04	48	8.39	83	14.82	34	6.94	62	10.16	**326**	**10.11**
Veneto	71	8.53	70	7.78	78	8.26	119	12.03	51	6.30	80	10.51	**469**	**8.96**
Friuli Venezia Giulia	34	7.82	25	5.56	20	3.94	52	9.61	23	4.73	41	8.17	**195**	**6.68**
Liguria	33	7.37	36	7.71	43	8.96	48	12.03	27	6.07	43	10.67	**230**	**8.71**
Emilia Romagna	26	5.44	34	7.16	41	8.17	57	10.59	26	6.15	65	11.42	**249**	**8.34**
Tuscany	25	5.43	35	7.22	23	4.41	63	11.98	24	4.88	44	9.67	**214**	**7.28**
Umbria	12	6.86	10	5.71	19	9.22	27	13.78	16	7.51	20	7.52	**104**	**8.45**
Marche	46	9.33	50	11.21	24	4.49	61	12.58	22	4.78	47	8.74	**250**	**8.46**
Latium	34	7.00	35	7.49	28	6.14	56	11.69	23	4.42	53	12.86	**229**	**8.12**
Abruzzo	42	9.46	37	8.56	31	6.71	62	15.27	21	4.58	56	10.65	**249**	**9.12**
Molise	35	9.36	39	10.46	35	7.1	57	13.73	27	7.44	34	9.16	**227**	**9.5**
Campania	49	10.72	51	13.49	42	8.96	73	15.73	23	5.08	48	10.43	**286**	**10.67**
Apulia	37	8.22	45	9.98	42	8.11	79	16.42	40	8.02	76	14.18	**319**	**10.87**
Basilicata	19	6.96	24	10.26	22	7.83	40	12.31	17	6.59	16	8.00	**138**	**8.78**
Calabria	42	8.96	42	10.07	37	7.13	71	13.71	33	7.28	60	11.88	**285**	**9.89**
Sicily	41	10.00	40	9.85	50	10.37	67	14.14	23	4.38	52	11.71	**273**	**9.96**
Sardinia	20	8.40	26	9.89	21	6.29	36	12.24	9	5.73	19	10.73	**131**	**8.95**
Bolzano	28	8.86	27	7.83	24	6.49	49	12.22	31	10.16	32	8.94	**191**	**9.12**
Trento	43	10.34	33	7.73	44	8.78	46	9.22	27	5.72	37	7.06	**230**	**8.1**
**Total**	**736**	**8.26**	**759**	**8.61**	**721**	**7.25**	**1242**	**12.71**	**526**	**5.86**	**938**	**10.19**	**4922**	**8.85**
